# Beyond Spirometry: Dyspnea, Functional Limitation, and Psychological Distress Across GOLD Stages in COPD—A Multicenter Observational Study

**DOI:** 10.3390/medicina62081500

**Published:** 2026-08-04

**Authors:** Adina Deliu, Luana Alexandrescu, Bogdan Cimpineanu, Oana Cristina Arghir, Sanda Jurja, Ioan Tiberiu Tofolean, Rodica Gabriela Enache, Ioana Gherghisan, Ionela Preotesoiu, Ionut Valentin Stanciu, Andreea Nelson Twakor, Alexandra Herlo, Daria Maria Alexandrescu, Doina Ecaterina Tofolean

**Affiliations:** 1 Doctoral School, Faculty of General Medicine, “Ovidius” University, 900470 Constanta, Romania; adina.deliu@365.univ-ovidius.ro (A.D.); oana.arghir@365.univ-ovidius.ro (O.C.A.); sanda.jurja@365.univ-ovidius.ro (S.J.); ioana.husaru@gmail.com (I.G.); ionela.preotesoiu@365.univ-ovidius.ro (I.P.); ionutstvalentin@gmail.com (I.V.S.); doina.tofolean@365.univ-ovidius.ro (D.E.T.); 2 Faculty of General Medicine, “Ovidius” University, 900470 Constanta, Romania; bogdan.cimpineanu@365.univ-ovidius.ro (B.C.); ioan.tofolean@365.univ-ovidius.ro (I.T.T.); 3 Gastroenterology Department, “Sf. Apostol Andrei” Emergency County Hospital, 145 Tomis Blvd., 900591 Constanta, Romania; 4 Department of Internal Medicine, “Sf. Apostol Andrei” Emergency County Hospital, 145 Tomis Blvd., 900591 Constanta, Romania; andreea.purcaru@365.univ-ovidius.ro; 5 Nephrology Department, “Sf. Apostol Andrei” Emergency County Hospital, 145 Tomis Blvd., 900591 Constanta, Romania; 6 Faculty of Psychology and Educational Sciences, “Ovidius” University, 900470 Constanta, Romania; rodica-gabriela.enache@365.univ-ovidius.ro; 7 Pneumology Department, Pneumophtisiology Hospital Palazu Mare, 40 Santinelei Street, 900002 Constanta, Romania; 8 Pneumology Department, “Sf. Apostol Andrei” Emergency County Hospital, 145 Tomis Blvd., 900591 Constanta, Romania; 9 Department XIII, Discipline of Infectious Diseases, “Victor Babes” University of Medicine and Pharmacy Timisoara, 2 Eftimie Murgu Square, 300041 Timisoara, Romania; alexandra.mocanu@umft.ro; 10 Faculty of Medicine, Titu Maiorescu University, 040051 Bucharest, Romania

**Keywords:** COPD, GOLD stage, anxiety, depression, dyspnea, psychological distress, functional limitation, pulmonary function, quality of life, respiratory disease

## Abstract

*Background*: Chronic obstructive pulmonary disease (COPD) is frequently accompanied by psychological distress, but the relationship between GOLD stage, dyspnea, functional limitation, and depression/anxiety symptoms remains incompletely defined. This study examined clinical and psychological profiles across GOLD stages, with particular attention to GOLD stages 3 and 4. *Methods*: This multicenter observational study included 285 adults with spirometry-confirmed COPD evaluated in Romania between 2023 and 2026. COPD severity was classified according to GOLD stages. Clinical assessment included FEV1, peripheral oxygen saturation, smoking exposure, the COPD Assessment Test (CAT), and the modified Medical Research Council dyspnea scale (mMRC). Psychological and well-being measures included DASS depression, anxiety, and stress scores; WHO-5; major depressive disorder score, and generalized anxiety disorder score. Descriptive analyses were performed across GOLD stages, and GOLD 3 was directly compared with GOLD 4. *Results*: GOLD 3 was the largest subgroup (*n* = 106, 37.2%), followed by GOLD 4 (*n* = 81, 28.4%), GOLD 2 (*n* = 69, 24.2%), and GOLD 1 (*n* = 29, 10.2%). GOLD 3 patients showed marked dyspnea and symptom burden, with mean mMRC = 2.92 and CAT = 24.44. DASS depression, anxiety, and stress scores were higher in GOLD 3 than GOLD 2, but did not increase further in GOLD 4. Direct GOLD 3 versus GOLD 4 comparisons showed no significant differences in DASS depression or DASS anxiety, whereas GOLD 4 had a significantly higher mMRC, major depressive disorder score, and generalized anxiety disorder score. *Conclusions*: GOLD 3 COPD was associated with substantial dyspnea, functional limitation, and measurable psychological distress. However, depression and anxiety patterns varied by instrument, suggesting that psychological burden in COPD is multidimensional and not explained by spirometric severity alone.

## 1. Introduction

Chronic obstructive pulmonary disease is a progressive respiratory disorder characterized by persistent airflow limitation, dyspnea, reduced exercise tolerance, and recurrent exacerbations, with systemic consequences that extend beyond pulmonary function alone [1]. Contemporary concepts propose that the traditional spirometry-based definition of COPD may not fully capture the complexity of the disease, highlighting the contribution of defective lung regeneration and structural remodeling to disease development and progression [1,2]. In recent years, psychological distress has been increasingly recognized as a clinically relevant comorbidity in COPD, particularly anxiety and depression, which may worsen symptom perception, self-management, treatment adherence, physical activity, and prognosis [3,4,5]. These symptoms are not only frequent in advanced COPD but may also appear in mild and moderate disease, suggesting that psychological burden can develop across the full clinical spectrum [3].

The relationship between COPD and mental health is complex and bidirectional. Dyspnea, functional limitation, social isolation, sleep disturbance, and fear of exacerbation may contribute to depressive and anxious symptoms, while anxiety and depression can intensify perceived breathlessness, reduce participation in pulmonary rehabilitation, and negatively affect adherence to therapy [3,4,5,6]. Several interventions, including psychological support, tele-based care, pulmonary rehabilitation, exercise training, palliative approaches, and integrated disease management, have therefore been proposed to improve both emotional symptoms and quality of life in COPD [7,8,9].

Despite this growing evidence, the association between COPD severity and psychological distress remains inconsistent. Some studies suggest that anxiety and depression increase with disease burden, exacerbation risk, and functional impairment, whereas others indicate that spirometric severity alone does not fully explain psychological outcomes [10,11,12]. This distinction is particularly relevant in severe COPD, where dyspnea, symptom burden, and functional limitation may provide more clinically meaningful information than FEV1 alone. Therefore, the present study investigates dyspnea, functional limitation, and psychological distress in COPD, with particular attention to GOLD stage 3 and its relationship with depression and anxiety measures.

## 2. Materials and Methods

### 2.1. Study Design and Setting

This multicenter observational study included patients with COPD evaluated between April 2023 and March 2026 in two clinical centers in Constanța, Romania: Constanța Pneumophtisiology Hospital “Palazu Mare” and County Emergency Hospital “Sf. Apostol Andrei” Constanța (Medical Clinic 1 and Medical Clinic 2). The final study sample consisted of 285 adults with spirometry-confirmed COPD. All data were obtained from routine clinical records and standardized assessments performed during usual care, without additional protocol-driven interventions.

The study was conducted in accordance with the Declaration of Helsinki and received approval from the ethics committees of the participating institutions, under protocol codes 2400/21 March 2023 and 24907/26 April 2023.

### 2.2. Participants and Eligibility Criteria

Patients were included if they were aged 18 years or older and had a confirmed diagnosis of COPD based on GOLD criteria, defined by a post-bronchodilator FEV1/FVC ratio below 0.70. Only clinically stable patients at the time of evaluation were considered eligible.

Patients were excluded if they had another primary respiratory diagnosis, were assessed during an acute COPD exacerbation, had severe cognitive impairment that could compromise questionnaire validity, or had incomplete data for the key clinical or psychological variables required for analysis.

### 2.3. Clinical and Functional Assessment

COPD severity was classified according to GOLD spirometric stages using post-bronchodilator FEV1 percentage of predicted value. Patients were categorized as GOLD 1, GOLD 2, GOLD 3, or GOLD 4. Respiratory evaluation included FEV1, FVC, FEV1/FVC ratio, and peripheral O_2_ saturation at rest. Smoking exposure was recorded using smoking status and cumulative pack-year index.

Symptom burden and functional limitation were assessed using CAT score and the modified mMRC. These variables were included to characterize the relationship between respiratory severity, dyspnea, daily functional limitation, and psychological distress.

### 2.4. Psychological Assessment

We evaluated psychological distress using depression, anxiety, and stress scores from the DASS scale. Well-being was assessed using the WHO-5 index. Additional disorder-oriented psychological measures included major depressive disorder and generalized anxiety disorder scores, which were analyzed as continuous variables. The main focus of the analysis was the relationship between COPD severity, particularly GOLD stage 3, and psychological distress indicators. The Romanian-language versions of the DASS and the WHO-5 used in routine clinical practice have undergone translation and cultural adaptation for Romanian populations. In the present study, these instruments were administered as part of standard clinical assessment, and the recorded scores were extracted from medical records. Because this investigation relied on routinely collected clinical data, no additional psychometric validation or internal consistency analyses were performed within the study cohort.

### 2.5. Statistical Analysis

We summarized continuous variables as means, standard deviations, and 95% confidence intervals. We also reported categorical variables as frequencies and percentages.

Descriptive comparisons were performed across GOLD stages 1 to 4. We paid attention to GOLD stage 3 because it represented the largest subgroup and corresponded to severe COPD. GOLD 3 was also compared directly with GOLD 4 to evaluate whether psychological distress increased further in very severe COPD.

Normality was assessed using Kolmogorov–Smirnov and Shapiro–Wilk tests, together with graphical inspection of histograms and Q-Q plots. For comparisons between GOLD 3 and GOLD 4, independent-samples t-tests were used. When equality of variances was not met according to Levene’s test, Welch’s correction was applied. Effect sizes were reported using Hedges’ g.

Principal component analysis (PCA) with Varimax orthogonal rotation was performed to explore the underlying relationships among psychological, clinical, and functional variables. Sampling adequacy was assessed using the Kaiser–Meyer–Olkin (KMO) measure, and Bartlett’s test of sphericity was used to confirm the suitability of the data for factor analysis. Components were retained according to the Kaiser criterion (eigenvalues > 1). Rotated factor loadings ≥0.40 were considered meaningful for interpretation, whereas lower loadings were suppressed from the rotated component matrix to improve interpretability. Statistical significance was defined as *p* < 0.05. All analyses were performed using IBM SPSS Statistics version 30.0 (IBM Corp., Armonk, NY, USA).

## 3. Results

### 3.1. Sample Structure and Completeness of Data

A total of 285 patients were included in the analysis, with complete data available for all variables entered in the GOLD-stratified analyses. The distribution across COPD severity stages was uneven, with GOLD 3 representing the largest subgroup. Specifically, 29 patients were classified as GOLD 1 (10.2%), 69 as GOLD 2 (24.2%), 106 as GOLD 3 (37.2%), and 81 as GOLD 4 (28.4%). Thus, 187 patients (65.6%) were classified as having severe or very severe COPD according to GOLD stage 3 or 4.

The GOLD 3 subgroup was therefore central to the clinical profile of the cohort, both because it represented the largest individual stage and because it marked the transition between moderate disease burden and the more advanced clinical phenotype observed in GOLD 4 (Table 1).

Across GOLD stages, the most consistent clinical gradient was observed for dyspnea and cumulative tobacco exposure. Mean mMRC score increased from 0.90 in GOLD 1 to 1.67 in GOLD 2, 2.92 in GOLD 3, and 3.53 in GOLD 4. The pack-year index followed a similarly ordered pattern, rising from 9.59 in GOLD 1 to 28.38 in GOLD 2, 38.89 in GOLD 3, and 52.94 in GOLD 4.

FEV1 and CAT scores did not show a strictly monotonic pattern across all four stages. Mean FEV1 was 39.8 in GOLD 3 and 24.6 in GOLD 4. CAT scores were high across all stages, with the highest mean observed in GOLD 3 (24.44), followed by GOLD 4 (23.90), GOLD 1 (23.52), and GOLD 2 (21.30). Resting oxygen saturation showed a modest downward pattern in the advanced stages, with mean SpO_2_ decreasing from 94.16% in GOLD 2 to 92.24% in GOLD 3 and 91.62% in GOLD 4. Table S1 includes the full case processing summary. 

**Figure 1:**
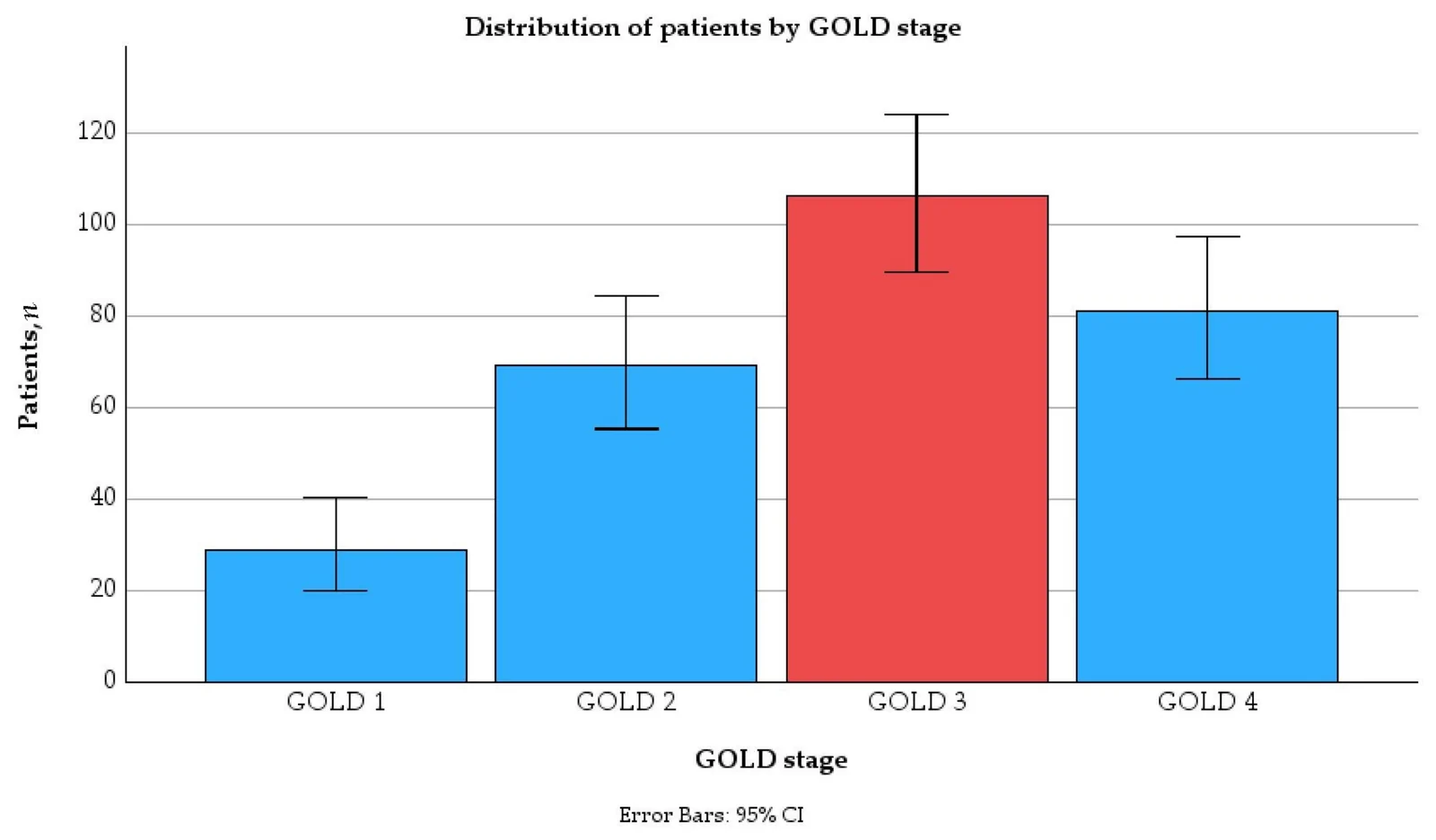
Distribution of patients by GOLD stage. Bar chart showing the number and percentage of patients in GOLD 1 (*n* = 29; 10.2%), GOLD 2 (*n* = 69; 24.2%), GOLD 3 (*n* = 106; 37.2%), and GOLD 4 (*n* = 81; 28.4%). GOLD 3 is highlighted as the largest subgroup.

Figure 1 shows the distribution of patients by GOLD stage.

### 3.2. Psychological Distress and Well-Being Across GOLD Stages

Psychological indicators were examined across GOLD stages using DASS depression, DASS anxiety, DASS stress; WHO-5; major depressive disorder score (MDD), and generalized anxiety disorder (GAD) scores from PDSQ. The DASS depression and DASS anxiety scores were higher in GOLD 3 compared with GOLD 2, but they did not increase further in GOLD 4. Mean DASS depression was 1.48 in GOLD 2, 1.90 in GOLD 3, and 1.81 in GOLD 4. Mean DASS anxiety was 1.55 in GOLD 2, 1.92 in GOLD 3, and 1.95 in GOLD 4. Thus, GOLD 3 was associated with higher mean DASS depression and anxiety scores than GOLD 2, but the GOLD 3 and GOLD 4 values were closely similar.

By contrast, the MDD and GAD scores displayed a clearer stage-related increase across advanced COPD categories. The mean MDD score increased from 2.59 in GOLD 1 to 3.19 in GOLD 2, 3.90 in GOLD 3, and 5.12 in GOLD 4. The mean GAD score increased from 1.97 in GOLD 1 to 3.23 in GOLD 2, 3.70 in GOLD 3, and 4.64 in GOLD 4. This pattern positions GOLD 3 as an intermediate but clinically important stage in the progression of depression- and anxiety-related symptom burden, with the highest values observed in GOLD 4 (Table 2).

Within the severe COPD subgroup, GOLD 3 patients showed a psychological profile characterized by moderate symptom elevation and broad overlap with GOLD 4 on the DASS subscales. The mean DASS depression score in GOLD 3 was 1.90, compared with 1.81 in GOLD 4. The mean DASS anxiety score in GOLD 3 was 1.92, compared with 1.95 in GOLD 4.

**Figure 2:**
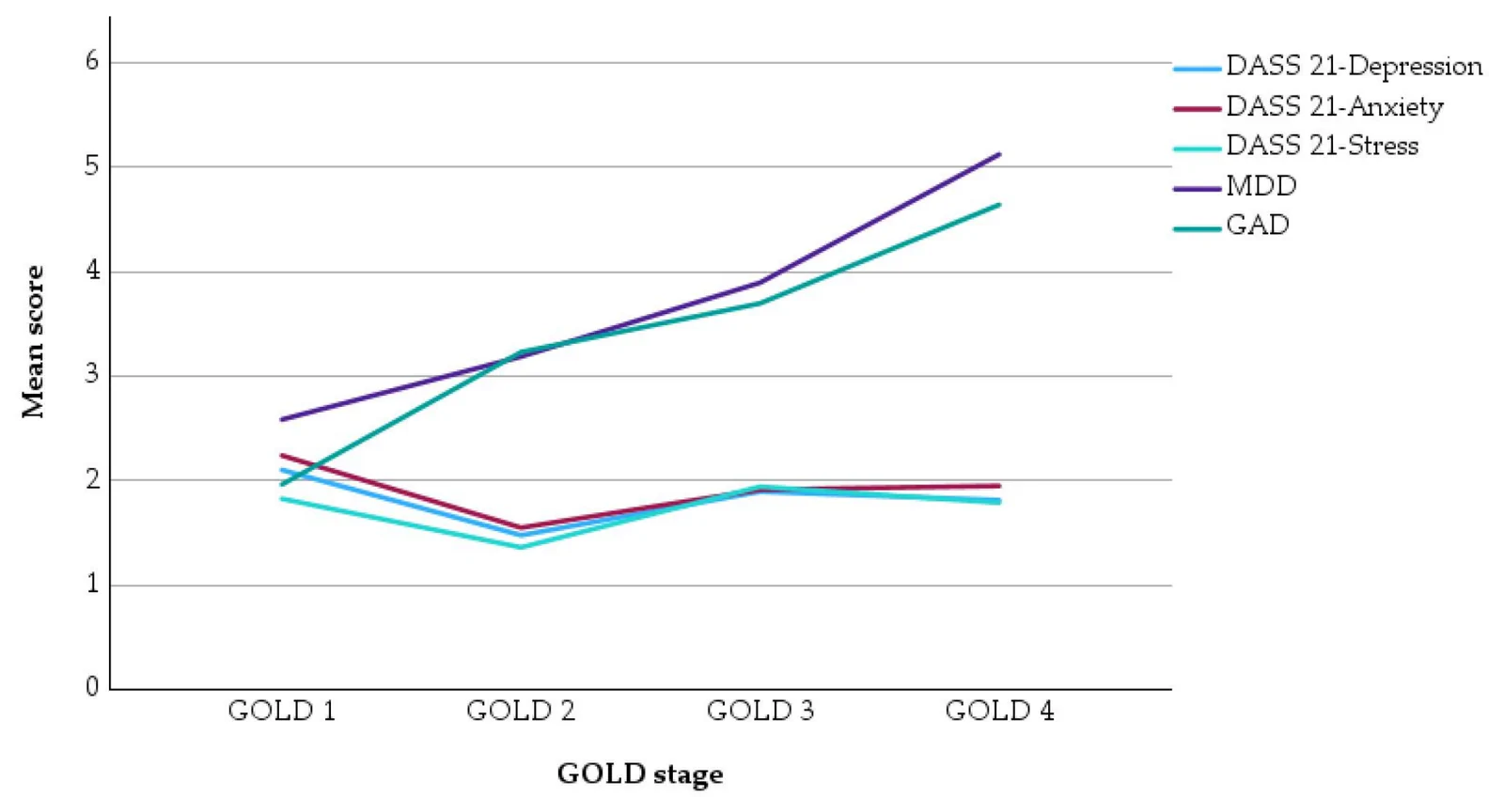
Psychological distress profile across GOLD stages. Multi-panel line or grouped-bar figure showing mean DASS depression, DASS anxiety, DASS stress, MDD score, and GAD score by GOLD stage.

However, depression and anxiety indicators based on the MDD and GAD variables increased between GOLD 3 and GOLD 4. The MDD score increased from 3.90 in GOLD 3 to 5.12 in GOLD 4, while the GAD score increased from 3.70 in GOLD 3 to 4.64 in GOLD 4. Figure 2 shows the psychological distress across GOLD stages.

### 3.3. GOLD 3 Versus GOLD 4 Comparison

Because the study objective emphasized the relationship between psychological distress and severe COPD, direct comparisons were performed between GOLD 3 and GOLD 4. These comparisons showed that GOLD 3 and GOLD 4 were similar in SpO_2_, DASS depression, and DASS anxiety, whereas GOLD 4 had significantly lower FEV1 values and significantly higher mMRC, MDD and GAD scores, and lower WHO-5 score.

For DASS depression, the GOLD 3 mean was 1.90 and the GOLD 4 mean was 1.81. The between-group difference was not statistically significant, t(185) = 0.515, *p* = 0.607, mean difference = 0.081, and 95% CI was −0.230 to 0.393. The effect size was negligible (Hedges g = 0.076). For DASS anxiety, the GOLD 3 mean was 1.92 and the GOLD 4 mean was 1.95. This difference was also not statistically significant, t(185) = −0.215, *p* = 0.830, mean difference = −0.036, and 95% CI was −0.362 to 0.291, with a negligible effect size (Hedges g = −0.032).

In contrast, MDD scores were significantly higher in GOLD 4 than GOLD 3. The mean score was 3.90 in GOLD 3 and 5.12 in GOLD 4, corresponding to a mean difference of −1.227 when calculated as GOLD 3 minus GOLD 4. This difference was statistically significant, t(185) = −3.668, *p* < 0.001, and 95% CI was −1.887 to −0.567, with a moderate effect size (Hedges g = −0.539). GAD scores were also significantly higher in GOLD 4 than GOLD 3. The mean score was 3.70 in GOLD 3 and 4.64 in GOLD 4, with a mean difference of −0.944, t(185) = −2.663, *p* = 0.008, 95% CI of −1.643 to −0.245, and a small-to-moderate effect size (Hedges g = −0.391).

The respiratory and functional comparisons supported a stronger difference between GOLD 3 and GOLD 4 for dyspnea than for spirometric or oxygenation variables. Mean mMRC was 2.92 in GOLD 3 and 3.53 in GOLD 4, and this difference was statistically significant using the unequal-variance test, t(176.522) = −7.993, *p* < 0.001, mean difference = −0.606, and 95% CI was −0.756 to −0.457. The effect size was large (Hedges g = −1.167). Conversely, FEV1 differed significantly between GOLD 3 and GOLD 4. Mean FEV1 was substantially lower in GOLD 4 (24.6 ± 4.84) compared with GOLD 3 (39.8 ± 5.41), t(185) = 19.98, *p* < 0.001, mean difference = 15.20, and 95% CI was 13.70 to 16.70, with a very large effect size (Hedges g = 2.95). SpO_2_ also did not differ significantly between GOLD 3 and GOLD 4, t(182.328) = 0.969, *p* = 0.334, mean difference = 0.619 percentage points, and 95% CI was −0.641 to 1.878 (Table 3).

**Figure 3:**
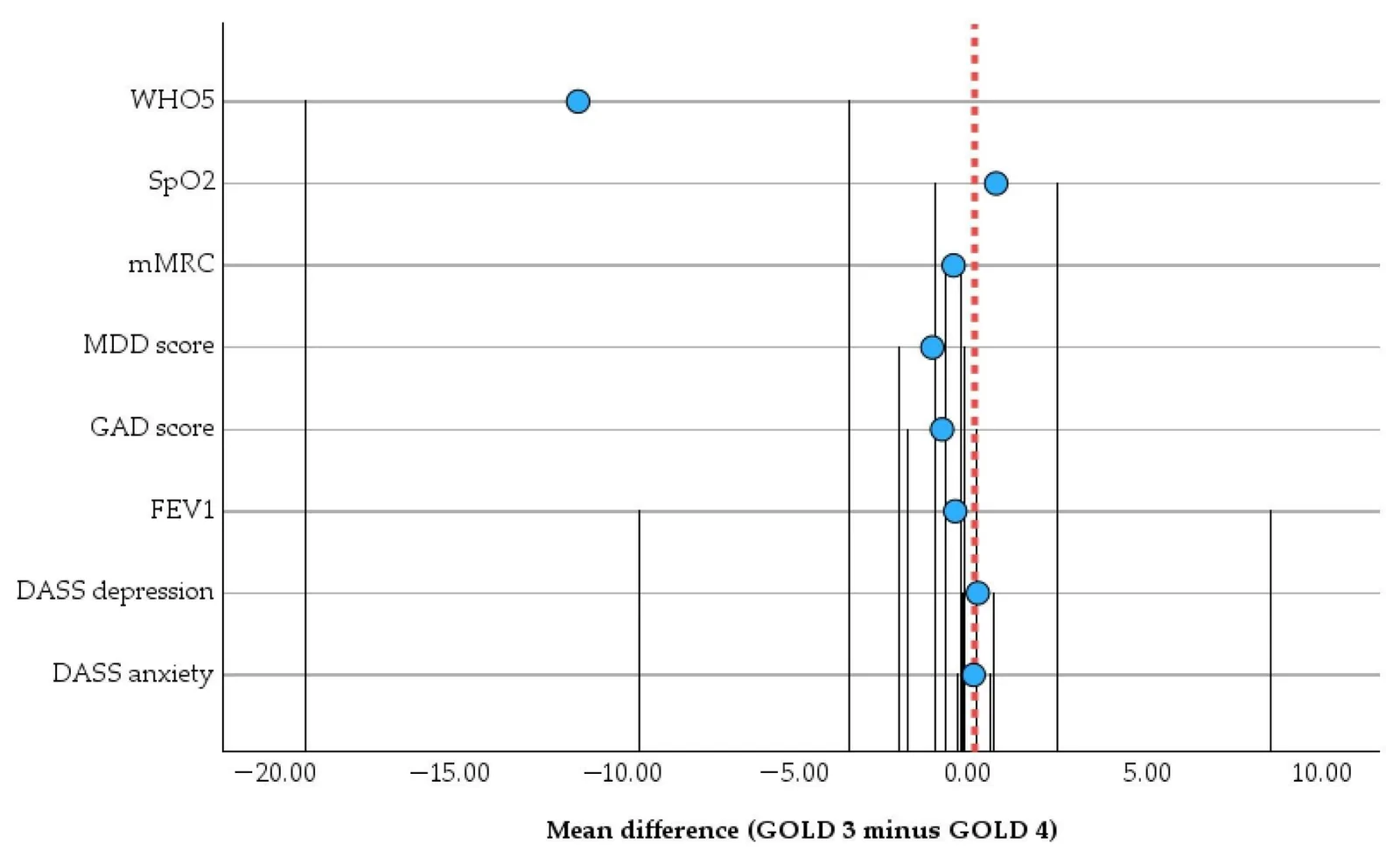
GOLD 3 versus GOLD 4 comparison for clinical and psychological outcomes. Forest plot of mean differences calculated as GOLD 3 minus GOLD 4 with 95% confidence intervals.

The GOLD 3 versus GOLD 4 analysis indicates that the direct association between advanced COPD stage and DASS-defined depression or anxiety was weak in this cohort. The data do not support a statistically significant difference in DASS depression or DASS anxiety between GOLD 3 and GOLD 4 (Figure 3). Nevertheless, GOLD 3 was not psychologically unaffected: it showed elevated symptom levels compared with GOLD 2 descriptively, and it was associated with substantial dyspnea, high CAT scores, reduced SpO_2_ relative to earlier stages, and increased MDD and GAD scores compared with GOLD 1 and GOLD 2. The strongest statistically significant differences between GOLD 3 and GOLD 4 were observed for FEV1, mMRC, WHO-5, MDD score, and GAD score.

### 3.4. Relationship Between Symptom Burden, Functional Limitation, and Psychological Distress

The covariance structure among psychological, respiratory, and functional variables indicated that psychological distress clustered with symptom burden rather than with spirometric separation between GOLD 3 and GOLD 4. DASS depression and DASS anxiety were strongly related to each other and loaded together with CAT on the first extracted principal component. In the rotated component matrix, DASS anxiety had the highest loading on Component 1 (0.896), followed by DASS depression (0.859) and CAT (0.730). This component can be interpreted as a psychological-symptom burden dimension, combining affective distress with patient-reported respiratory impact.

The second extracted component was defined primarily by mMRC, MDD score, GAD score, and WHO-5. Rotated loadings were 0.737 for mMRC, 0.725 for MDD score, 0.665 for GAD score, and 0.726 for WHO-5. This pattern suggests that dyspnea-related functional limitation was more closely aligned with disorder-oriented depression and anxiety scores than with the brief DASS depression and anxiety subscales.

The factor solution was acceptable for exploratory interpretation. The Kaiser–Meyer–Olkin measure of sampling adequacy was 0.586, and Bartlett’s test of sphericity was statistically significant, χ^2(21)^ = 470.031, *p* < 0.001. Two components had eigenvalues greater than 1 and together explained 56.595% of total variance. Component 1 explained 30.841% of the variance, and Component 2 explained 25.754%. The separation of the DASS-CAT component from the mMRC-disorder score component indicates that psychological distress in COPD was not captured by a single uniform construct in this sample.

The two-component solution revealed distinct but complementary dimensions of COPD-related symptomatology and psychological burden. Component 1 was characterized by high loadings for DASS depression, DASS anxiety, and CAT score, suggesting a psychological symptom burden dimension that integrates emotional distress with patient-reported respiratory symptom impact. In contrast, Component 2 was defined primarily by mMRC dyspnea, MDD score, GAD score, and WHO-5, representing a functional impairment and disorder-oriented psychological distress dimension. This pattern indicates that functional limitation and disorder-specific measures of anxiety and depression clustered separately from the broader emotional distress captured by the DASS subscales (Table 4).

**Figure 4:**
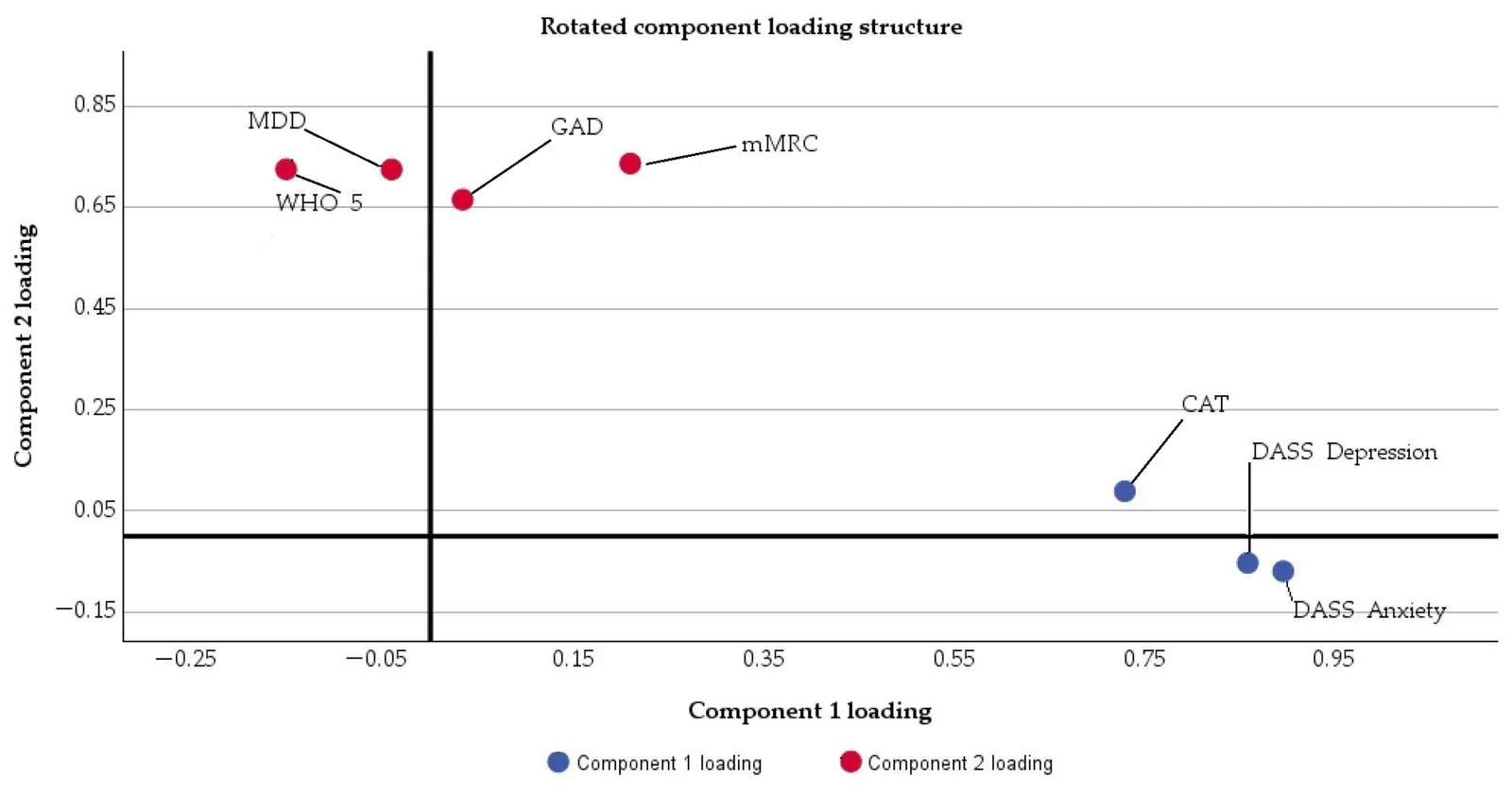
Principal component structure of distress and functional limitation.

The factor findings support a nuanced interpretation of the GOLD 3 results. GOLD 3 showed a high CAT score and elevated DASS depression, anxiety, and stress scores relative to GOLD 2, suggesting that psychological distress was already present at the severe COPD stage. However, the transition from GOLD 3 to GOLD 4 was most clearly expressed through dyspnea-related functional limitation and higher scores on the MDD and GAD variables. Thus, GOLD 3 appears to represent a clinically relevant stage in which respiratory symptom impact and psychological distress coexist, but the strongest statistical differentiation from GOLD 4 was not observed on the DASS depression and anxiety subscales (Figure 4).

The cohort was predominantly composed of patients with advanced COPD, with GOLD 3 representing the largest stage subgroup and GOLD 3 plus GOLD 4 accounting for nearly two thirds of the sample. GOLD 3 was characterized by substantial dyspnea, high CAT score, reduced oxygen saturation compared with earlier stages, and increased psychological symptom burden compared with GOLD 2 on several descriptive indicators. The GOLD 3 subgroup therefore represents a clinically important point at which respiratory burden and psychological distress overlap.

The direct GOLD 3 versus GOLD 4 comparison showed little evidence of a statistically significant difference in DASS depression or DASS anxiety. These two DASS subscales were nearly equivalent across the two advanced GOLD stages. However, GOLD 4 had significantly higher mMRC, MDD and GAD scores, and a lower WHO-5 score than GOLD 3. Thus, the relationship between COPD severity and psychological distress was present but instrument-dependent. The strongest evidence of worsening psychological distress from GOLD 3 to GOLD 4 was observed for MDD and gGAD scores, while DASS depression and DASS anxiety appeared to plateau across the severe and very severe COPD categories.

Our results support an association between advanced COPD, functional limitation, and psychological distress, but they do not support a simple linear worsening of all psychological measures from GOLD 3 to GOLD 4. Instead, GOLD 3 appears to be a key severe-disease stage marked by high symptom burden and measurable distress, while GOLD 4 is distinguished most clearly by greater dyspnea and higher disorder-oriented depression and anxiety scores.

## 4. Discussion

The present findings show that GOLD stage 3 represented the largest subgroup and was characterized by substantial dyspnea, high CAT scores, reduced oxygen saturation compared with earlier stages, and measurable psychological distress. This is consistent with the literature describing anxiety and depression as common comorbidities that increase the clinical and economic burden of COPD [5]. However, our results also showed that the relationship between COPD severity and psychological distress was not uniform across instruments. DASS depression and DASS anxiety were elevated in GOLD 3 compared with GOLD 2 but did not differ significantly between GOLD 3 and GOLD 4. This partially agrees with studies suggesting that psychological symptoms are influenced by modifiable factors, functional status, and symptom perception rather than by airflow limitation alone [13,14].

The strongest GOLD 3 to GOLD 4 differences in our study were observed for FEV1, mMRC, MDD and GAD scores, and WHO-5. While spirometric decline clearly differentiated GOLD 3 from GOLD 4, DASS depression and anxiety scores remained relatively stable between these advanced stages. These findings align with evidence that dyspnea, poor physical activity, and impaired daily functioning are closely linked with anxiety and depression in severe COPD [6]. The significant increase in disorder-oriented depression and anxiety scores from GOLD 3 to GOLD 4 also supports registry-based and primary care findings showing a high prevalence of anxiety/depression in COPD, particularly in patients with greater disease burden or vulnerability [15,16,17]. At the same time, the absence of significant GOLD 3 versus GOLD 4 differences in DASS depression and anxiety suggests a possible plateau effect, or that brief symptom scales may capture distress differently from diagnostic screening tools.

We also highlight the importance of integrated and non-pharmacological care. Evidence syntheses indicate that pulmonary rehabilitation, nursing care, telecare, non-invasive ventilation support, and structured disease-management programs may improve quality of life and emotional outcomes in COPD [18,19,20,21,22]. Qualitative research from TANDEM also highlights that psychological care must be acceptable, tailored, and embedded within respiratory care pathways [23].

In this context, GOLD 3 may represent an important intervention window: patients already show marked functional limitation and psychological burden, but before the more pronounced distress observed in GOLD 4.

Our study agrees with the literature that COPD-related psychological distress is multidimensional. Depression and anxiety should not be interpreted only as consequences of spirometric decline, but as outcomes shaped by dyspnea, functional limitation, quality of life, living environment, and disease management needs.

Psychological distress in COPD is a multifactorial phenomenon that extends beyond the degree of airflow limitation or symptom burden. Previous studies have demonstrated that factors such as social support, socioeconomic status, medical comorbidities, medication use, and access to healthcare may substantially influence the development and severity of anxiety and depressive symptoms [24,25].

One of the limitations of this study relates to the psychological assessment instruments. Although the Romanian-language versions of the DASS and WHO-5 have undergone translation and cultural adaptation, the design of this study did not permit an independent evaluation of their psychometric properties within the present cohort. Consequently, measures of internal consistency, construct validity, and test–retest reliability were not assessed. Future prospective studies should include formal psychometric evaluation of these instruments in Romanian patients with COPD to further strengthen the interpretation of psychological outcomes.

Although all patients included in the final analyses had complete data for the variables of interest, the nature of the study limited the availability of potentially relevant clinical and psychosocial variables, including socioeconomic status, social support, medication use, and comorbidities, which may have influenced psychological outcomes and introduced residual confounding.

Also, although the study was conducted in two tertiary referral centers using similar clinical protocols and standardized assessment procedures, inter-center variability in patient characteristics, referral patterns, and routine clinical practice cannot be completely excluded. Such differences may have influenced some clinical and psychological measurements and should be considered when interpreting the results.

## 5. Conclusions

This multicenter study showed that psychological distress is closely intertwined with dyspnea and functional limitation in patients with COPD, particularly among those classified as GOLD stage 3. GOLD 3 represented the largest subgroup and was characterized by high CAT scores, marked mMRC dyspnea, reduced oxygen saturation compared with earlier stages, and increased depression, anxiety, and stress scores compared with GOLD 2. Thus, GOLD 3 is a clinically important stage in which respiratory impairment and psychological vulnerability coexist.

However, the relationship between COPD severity and psychological distress was not linear across all measures. DASS depression and anxiety scores did not differ significantly between GOLD 3 and GOLD 4, indicating that brief symptom-based scales may plateau in advanced disease. In contrast, MDD and GAD scores were significantly higher in GOLD 4, alongside greater dyspnea burden.

Overall, the findings support routine psychological screening in patients with severe COPD, especially those with pronounced dyspnea and functional limitation. Integrating mental health assessment into COPD care may help identify vulnerable patients earlier and guide multidisciplinary interventions aimed at improving both respiratory outcomes and quality of life.

## Figures and Tables

**Table 1:** Clinical and respiratory characteristics according to GOLD stage.

Variable	GOLD 1 (*n* = 29)	GOLD 2 (*n* = 69)	GOLD 3 (*n* = 106)	GOLD 4 (*n* = 81)
Age, years, mean ± SD	67.86 ± 10.20	69.10 ± 9.13	64.60 ± 9.75	63.94 ± 8.53
FEV1, mean ± SD	86.4 ± 5.81	63.7 ± 8.12	39.8 ± 5.41	24.6 ± 4.84
SpO_2_, %, mean ± SD	93.76 ± 2.56	94.16 ± 3.18	92.24 ± 5.21	91.62 ± 3.51
CAT score, mean ± SD	23.52 ± 6.53	21.30 ± 8.44	24.44 ± 5.63	23.90 ± 5.86
mMRC score, mean ± SD	0.90 ± 0.56	1.67 ± 0.66	2.92 ± 0.53	3.53 ± 0.50
Pack-year index, mean ± SD	9.59 ± 8.77	28.38 ± 18.26	38.89 ± 18.22	52.94 ± 12.80

GOLD, Global Initiative for Chronic Obstructive Lung Disease; FEV1, forced expiratory volume in one second; SpO_2_, peripheral oxygen saturation; CAT, COPD Assessment Test; mMRC, modified Medical Research Council dyspnea scale; SD, standard deviation.

**Table 2:** Psychological distress and well-being indicators according to GOLD stage.

Variable	GOLD 1 (*n* = 29)	GOLD 2 (*n* = 69)	GOLD 3 (*n* = 106)	GOLD 4 (*n* = 81)
DASS depression, mean ± SD	2.10 ± 1.35	1.48 ± 1.31	1.90 ± 1.04	1.81 ± 1.11
DASS anxiety, mean ± SD	2.24 ± 1.24	1.55 ± 1.39	1.92 ± 1.10	1.95 ± 1.15
DASS stress, mean ± SD	1.83 ± 1.10	1.36 ± 1.12	1.94 ± 0.93	1.79 ± 1.03
WHO-5 score, %, mean ± SD	59.07 ± 20.61	47.63 ± 17.06	41.29 ± 18.50	23.38 ± 4.38
MDD score, mean ± SD	2.59 ± 2.47	3.19 ± 2.55	3.90 ± 2.20	5.12 ± 2.36
GAD score, mean ± SD	1.97 ± 1.18	3.23 ± 2.22	3.70 ± 2.27	4.64 ± 2.57

DASS, Depression Anxiety Stress Scales; WHO-5, World Health Organization–Five Well-Being Index; MDD, major depressive disorder score; GAD, generalized anxiety disorder score; GOLD, Global Initiative for Chronic Obstructive Lung Disease; SD, standard deviation.

**Table 3:** Independent-samples comparisons between GOLD 3 and GOLD 4.

Variable	GOLD 3 Mean ± SD	GOLD 4 Mean ± SD	Mean Difference	Test Statistic	Two-Sided *p*	95% CI of Difference	Hedges g
DASS depression	1.90 ± 1.04	1.81 ± 1.11	0.081	t(185) = 0.515	0.607	−0.230 to 0.393	0.076
DASS anxiety	1.92 ± 1.10	1.95 ± 1.15	−0.036	t(185) = −0.215	0.830	−0.362 to 0.291	−0.032
WHO-5 score, %	47.63 ± 17.06	23.38 ± 4.38	24.25	t(117.418) = 15.16	<0.001	−20.05 to 28.45	1.89
mMRC	2.92 ± 0.53	3.53 ± 0.50	−0.606	t(176.522) = −7.993	<0.001	−0.756 to −0.457	−1.167
SpO_2_, %	92.24 ± 5.21	91.62 ± 3.51	0.619	t(182.328) = 0.969	0.334	−0.641 to 1.878	0.135
FEV1	39.8 ± 5.41	24.6 ± 4.84	15.20	t(185) = 19.98	<0.001	13.70 to 16.70	2.95
MDD	3.90 ± 2.20	5.12 ± 2.36	−1.227	t(185) = −3.668	<0.001	−1.887 to −0.567	−0.539
GAD	3.70 ± 2.27	4.64 ± 2.57	−0.944	t(185) = −2.663	0.008	−1.643 to −0.245	−0.391

Note: Mean difference is calculated as GOLD 3 minus GOLD 4. Negative values therefore indicate higher values in GOLD 4. Unequal-variance results are reported for WHO-5, mMRC, SpO_2_, and FEV1 where appropriate based on Levene’s test; equal-variance results are reported for DASS depression, DASS anxiety, major depressive disorder score, and generalized anxiety disorder score.

**Table 4:** Rotated principal component matrix for psychological, symptom, and functional variables.

Variable	Component 1	Component 2
DASS anxiety	0.896	—
DASS depression	0.859	—
CAT	0.730	—
mMRC	—	0.737
MDD	—	0.725
GAD	—	0.665
WHO-5 score	—	0.726

Note: Principal component analysis with varimax rotation and Kaiser normalization. Loadings below 0.40 are suppressed. Rotation converged in three iterations. CAT, COPD Assessment Test; DASS, Depression Anxiety Stress Scales; GAD, generalized anxiety disorder score; MDD, major depressive disorder score; mMRC, modified Medical Research Council dyspnea scale; WHO-5, World Health Organization–Five Well-Being Index.

## Data Availability

Data is available on request to the corresponding author.

## Supplementary Materials

The following supporting information can be downloaded at https://www.mdpi.com/article/10.3390/medicina62081500/s1, Table S1: Case processing summary.

## Abbreviations

The following abbreviations are used in this manuscript:

COPD	Chronic obstructive pulmonary disease
GOLD	Global Initiative for Chronic Obstructive Lung Disease
FEV1	Forced expiratory volume in one second
FVC	Forced vital capacity
SpO_2_	Peripheral oxygen saturation
CAT	COPD assessment test
mMRC	Modified Medical Research Council dyspnea scale
DASS	Depression Anxiety Stress Scales
WHO-5	World Health Organization–5 Well-Being Index
MDD	Major depressive disorder
GAD	Generalized anxiety disorder
SD	Standard deviation
CI	Confidence interval
PCA	Principal component analysis
KMO	Kaiser–Meyer–Olkin
COPD Assessment Test	CAT
IBM SPSS	International Business Machines Statistical Package for the Social Sciences
OR	Odds ratio
COPD-QoL	Chronic obstructive pulmonary disease quality of life
BMI	Body mass index
ICU	Intensive care unit
Q-Q plot	Quantile–quantile plot
COPD exacerbation	Acute worsening of COPD symptoms
COPD-related distress	Psychological distress associated with COPD
