# Supplementary material for: Beyond Spirometry: Dyspnea, Functional Limitation, and Psychological Distress Across GOLD Stages in COPD—A Multicenter Observational Study
Source: Medicina (Kaunas). 2026 Aug 4;62(8):1500. doi: 10.3390/medicina62081500 (PMC13514793; doi:10.3390/medicina62081500)
Supplement: Supplementary file 1 [file medicina-62-01500-s001.zip › medicina-4451225-supplementary.pdf]

SUPPLEMENTARY MATERIAL

Table S1. Case processing summary

		Cases					
		Valid		Missing		Total	
	Stadiul GOLD	N	Percent	N	Percent	N	Percent
Age	GOLD 1	29	100.0%	0	0.0%	29	100.0%
	GOLD 2	69	100.0%	0	0.0%	69	100.0%
	GOLD 3	106	100.0%	0	0.0%	106	100.0%
	GOLD 4	81	100.0%	0	0.0%	81	100.0%
FEV1	GOLD 1	29	100.0%	0	0.0%	29	100.0%
	GOLD 2	69	100.0%	0	0.0%	69	100.0%
	GOLD 3	106	100.0%	0	0.0%	106	100.0%
	GOLD 4	81	100.0%	0	0.0%	81	100.0%
SpO2	GOLD 1	29	100.0%	0	0.0%	29	100.0%
	GOLD 2	69	100.0%	0	0.0%	69	100.0%
	GOLD 3	106	100.0%	0	0.0%	106	100.0%
	GOLD 4	81	100.0%	0	0.0%	81	100.0%
CAT	GOLD 1	29	100.0%	0	0.0%	29	100.0%
	GOLD 2	69	100.0%	0	0.0%	69	100.0%
	GOLD 3	106	100.0%	0	0.0%	106	100.0%
	GOLD 4	81	100.0%	0	0.0%	81	100.0%
mMRC	GOLD 1	29	100.0%	0	0.0%	29	100.0%
	GOLD 2	69	100.0%	0	0.0%	69	100.0%
	GOLD 3	106	100.0%	0	0.0%	106	100.0%
	GOLD 4	81	100.0%	0	0.0%	81	100.0%
DASS-Depression	GOLD 1	29	100.0%	0	0.0%	29	100.0%
	GOLD 2	69	100.0%	0	0.0%	69	100.0%
	GOLD 3	106	100.0%	0	0.0%	106	100.0%
	GOLD 4	81	100.0%	0	0.0%	81	100.0%
DASS-Anxiety	GOLD 1	29	100.0%	0	0.0%	29	100.0%
	GOLD 2	69	100.0%	0	0.0%	69	100.0%
	GOLD 3	106	100.0%	0	0.0%	106	100.0%
	GOLD 4	81	100.0%	0	0.0%	81	100.0%
DASS Stress	GOLD 1	29	100.0%	0	0.0%	29	100.0%
	GOLD 2	69	100.0%	0	0.0%	69	100.0%

	GOLD 3	106	100.0%	0	0.0%	106	100.0%
	GOLD 4	81	100.0%	0	0.0%	81	100.0%
WHO 5	GOLD 1	29	100.0%	0	0.0%	29	100.0%
	GOLD 2	69	100.0%	0	0.0%	69	100.0%
	GOLD 3	106	100.0%	0	0.0%	106	100.0%
	GOLD 4	81	100.0%	0	0.0%	81	100.0%
MDD	GOLD 1	29	100.0%	0	0.0%	29	100.0%
	GOLD 2	69	100.0%	0	0.0%	69	100.0%
	GOLD 3	106	100.0%	0	0.0%	106	100.0%
	GOLD 4	81	100.0%	0	0.0%	81	100.0%
GAD	GOLD 1	29	100.0%	0	0.0%	29	100.0%
	GOLD 2	69	100.0%	0	0.0%	69	100.0%
	GOLD 3	106	100.0%	0	0.0%	106	100.0%
	GOLD 4	81	100.0%	0	0.0%	81	100.0%
Pack-year index	GOLD 1	29	100.0%	0	0.0%	29	100.0%
	GOLD 2	69	100.0%	0	0.0%	69	100.0%
	GOLD 3	106	100.0%	0	0.0%	106	100.0%
	GOLD 4	81	100.0%	0	0.0%	81	100.0%
